# A Way to Increase the Bioaccesibility and Photostability of Roflumilast, a COPD Treatment, by Cyclodextrin Monomers

**DOI:** 10.3390/polym11050801

**Published:** 2019-05-04

**Authors:** Adrián Matencio, Samanta Hernández-García, Francisco García-Carmona, José Manuel López-Nicolás

**Affiliations:** Departamento de Bioquímica y Biología Molecular-A, Facultad de Biología, Universidad de Murcia—Regional Campus of International Excellence “Campus Mare Nostrum”, E-30100 Murcia, Spain; adrian.matencio@um.es (A.M.); samanta.hernandez@um.es (S.H.-G.); gcarmona@um.es (F.G.-C.)

**Keywords:** roflumilast, cyclodextrins, digestion, fluorescence, phosphodiesterase

## Abstract

Roflumilast is an orally available inhibitor of phosphodiesterase (PDE) type 4, which is widely used in chronic obstructive pulmonary diseases. However, it has low solubility and adverse effects include diarrhea and nausea. Since its solubilization may improve treatment and, dismissing any adverse effects, its interaction with cyclodextrins (CDs) was studied. The Higuchi-Connors method was used to determine the complexation constant with different CDs, pH values and temperatures. Molecular docking was used to predict interaction between the complexes. An in vitro digestion experiment was carried out to test roflumilast protection. Finally, the photostability of the complex was evaluated. The complex formed with β-CD had the highest *K*_11_ value (646 ± 34 M^−1^), although this value decreased with increasing temperature. Similarly, *K*_11_ decreased as the pH increased. In vitro digestion showed that CDs protect the drug during digestion and even improve its bioaccessibility. Finally, CDs reduced the drug’s extreme photosensitivity, originating a fluorescence signal, which is described for first time. The kinetic parameters of the reaction were obtained. This study not only completes the complexation study of roflumilast-CD, but also points to the need to protect roflumilast from light, suggesting that tablets containing the drug might be reformulated.

## 1. Introduction

**Figure 1:**
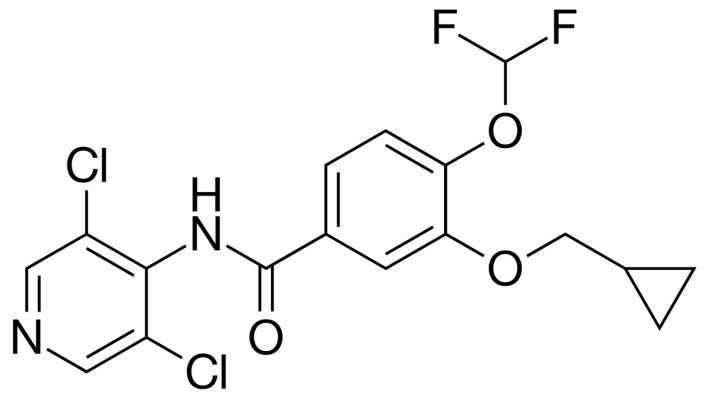
Structure of Roflumilast.

Roflumilast (Figure 1) is an orally available, long-acting inhibitor of phosphodiesterase (PDE) type 4 (PDE4), with anti-inflammatory and potential antineoplastic activities [1]. Roflumilast and its active metabolite roflumilast N-oxide are selective and competitive inhibitors of PDE4 [1], which leads to an increase in both the intracellular levels of cyclic-3′,5′-adenosine monophosphate (cAMP) and cAMP-mediated signaling. A recent review focused on its use in chronic obstructive pulmonary disease (COPD, [2]), for which it has been commonly administered since its approval in 2010 (EU) and 2011 (FDA).

Despite its good bioavailability, adverse effects such as diarrhea or nausea have been reported [3], its low solubility could originate an osmotic diarrhea [4] while the increase in concentration of cAMPprobably has the same effect [5]. Whatever the case, these observations suggest that treatment with roflumilast might improve if its solubility could be improved (and fewer drugs would be necessary). However, this bioactive molecule is easily oxidized and so any new strategy to improve its solubility and bioavailability must be able to limit any undesirable oxidation. In this paper, we analyze the encapsulation of roflumilast in a molecule with a known high complexation capacity: cyclodextrin (CD).

CDs are torus-shaped oligosaccharides made up of α-(1→4) linked glucopyranosideunits. The most common CDs are α, β and γ-CD, which contain six, seven and eight glucose units, respectively [6]. These types of natural CDs have two GRAS statuses and appear in the lists of additives approved for alimentary use with the corresponding E-numbers α-, β- and γ-CD: E-457, E-459 and E-458, respectively. The cavity of CDs is carpeted by hydrogen atoms and is therefore of an appreciably hydrophobic nature, unlike the outer surface of the molecule, in which the primary and secondary hydroxyl groups are exposed to the solvent, making the whole molecule highly water-soluble [6,7]. The complexes between molecules with CDs are called “inclusion complexes”. Although inorganic and organic salts and neutral molecules can form inclusion complexes with CDs [8], they are commonly known for the complexation of poorly water-soluble compounds and hydrophobic moieties of amphiphilic molecules, that are also highly water-soluble. However, the solubility of these complexes depends on several factors such as the type of CD used [9,10,11]. Because CDs are able to increase the bioavailability of different compounds and to protect different molecules against the impact of external agents, their use in both the pharmaceutical and food industries is continuously increasing [6,12,13].

In recent years our research group has published several works concerning the ability of CDs to encapsulate different molecules of the stilbene family [14,15,16] or fatty acids [17,18]. Recently, an article described how roflumilast was prepared with hydroxypropyl-β-CD (HPβ-CD), demonstrating that the CD can be a good carrier and that the resulting complex has a good apparent transepithelial permeability coefficient, similar to that of other drugs [19]. However, the study was only performed with HPβ-CD, overlooking a large number of CDs that might offer even better results. For example, no natural CDs were studied and, furthermore, the effect of temperature (a very important variable for release) was not studied.

Bearing the above in mind, the main objectives of this work were as follows:

(1) analyze the encapsulation mechanism of roflumilast by different types of natural and modified CDs.
(2) evaluate the effect of temperature and pH on the encapsulation mechanism of roflumilast.
(3) study the physical interactions between roflumilast and CDs using molecular docking.
(4) determine the stability and bioaccessibility(the quantity of a compound that is released from its matrix in the gastrointestinal tract, becoming available for absorption [20]) of the complex in digestion.
(5) understand the increased photostability of the complex.

## 2. Materials and Methods

### 2.1. Materials

α-, β- and γ-CD, pepsin-HCl, pancreatine and bilis were purchased from Sigma-Aldrich (Madrid, Spain). Hydroxypropyl-beta- and methyl-beta-cyclodextrin (HPβ-CD DS = 5 and Mβ-CD DS = 5.4) were purchased from Carbosynth (Berkshire, UK). Roflumilast (CID 5281717) was purchased from Xi An Kerui Biochemical CO (Xi’an, China) and used as received. The samples were stored in darkness. Ethanol (absolute, analysis grade) was purchased from Panreac (Madrid, Spain).

### 2.2. Equipment and Experimental Procedure

#### 2.2.1. Inclusion Complex Characterization

To characterize the encapsulation process, the method of Higuchi and Connors [21] was used. Tubes with specific quantities of CDs were prepared at a fixed quantity of roflumilast (8 mg/mL). Using different incubation times, it was demonstrated that a minimum of 10 h was necessary to achieve equilibrium, so, all the samples in this experiment were incubated for 12 h. A calibration curve was used to obtain roflumilast concentration (using roflumilast dissolved in 5% EtOH). 

This method is able to evaluate a 1:1 stoichiometry (1 CD per molecule). 

CD + roflumilast ⇆ CD-roflumilast(1)

Total solubility (S_t_) of the drug in solution with CDs was evaluated using the equation:

(2) $$S_{t}={\;S}_{0}+\frac{K_{F}S_{0}}{1+{\;K}_{F}S_{0}}\left[\mathrm{CD}\right]$$

where S_0_ is the intrinsic solubility of the drug, *K*_11_ is the apparent 1:1 complex stability constant and CD is the concentration of CD in the tube. Plotting the solubilized/complexed guest vs. solubilizer provides a “solubility isotherm” that can be fitted to Equation (2). This giving a slope that can be used to obtain K11 using the Higuchi and Connors method:

(3) $$K_{11}=\;\frac{Slope}{S_{0}\left(1-Slope\right)}$$

#### 2.2.2. Temperature and pH 

To study the effect of temperature on roflumilast encapsulation by CD, increasing temperatures of 278, 283, 288, 293, 298, 303, 310 and 318 K (5, 10, 15, 20, 25 30, 37 and 45 °C) were assayed. The thermodynamic relationship shown in Equation (3) was used to determine the standard thermodynamic parameters of enthalpy and entropy of roflumilast complexation in CD:

(4) $$Ln\;K_{11}=\;-\frac{\Delta H{}^{\circ}}{RT}+\frac{\Delta S{}^{\circ}}{R}$$

where *K*_11_ is the complexation constant of the inclusion complex, T is the temperature in Kelvin, R is the gas constant, Δ*H*° and Δ*S*° are the standard enthalpy and entropy changes of the complexes formed in the mobile phase. For a linear plot of ln *K*_11_ vs. 1/T, the slope and intercept were −ΔH°/R and ΔS°/R, respectively. To determine the Gibbs free energy change for the interactions that take place during the inclusion process, Equation (5) was used:

(5) ΔG°= ΔH°−TΔS°

For the pH studies, the same method as that described in the above section was followed at pH 6, 7.4 and 8 (although the incubation time was three hours due to the drug’s stability at pH 8). The following buffers were used i) pH (6–7.4) 100 mM Phosphate-Na and ii) pH 8 100 mM Borate-Na. 

#### 2.2.3. Molecular Docking

The molecular structures used in this work were obtained from several databases. β-CD was obtained from Protein Data Bank (ID 4RER) and used without modification. Roflumilast was downloaded from the PubChem database (NCBI, USA). HPβ-CD was build using Pymol (Molecular GraphicsSystem, version 1.3, Schrödinger, LLC) from β-CD. Input files for docking were generated using Autodock tools (version 1.5.6) with default parameters and charges. Molecular docking was carried out using AutodockVina [22] using default parameters. CDs were considered as flexible. A graphical representation of the docking result was prepared using PyMOL with default parameters to display hydrogen bonds.

#### 2.2.4. In Vitro Digestion

Three samples (i) control, (ii) roflumilast 0.2 mg/mL and (iii) roflumilast 0.2 mg/mL in the presence of 17.5 mg/mL of HPβ-CD (in a volume ingestion of 0.24 L) were subjected to the in vitro digestion model to assess the behavior of the complexes [23]. The protocol was the same as that described by Ilyasoglu (2014) with a few modifications: First, samples were prepared in saline at pH 3 with pepsin-HCl to simulate the gastric phase before incubating in a shaking water bath at 37 °C for 1 h. The incubation was stopped by adding 1 mol/m^3^ Na_2_CO_3_ to increase the pH to 6.9. In the subsequent intestinal phase, the pH was adjusted to 6.9 and a pancreatin–bile extract–lipase mixture was added before incubating at 37 °C for 2 h. Tubes were placed in an ice bath to stop digestion and centrifuged at 10,000× *g* at 4 °C for 35 min and the contents were diluted in phosphate-Na buffer pH 7.4 to achieve the same final concentration and filtered. The samples were analyzed using an Agilent HPLC 1200 series equipped with a TOF 6220 (acquisition range 100–1100) in negative mode. Six microliters of the soluble part were injected using 40/60 H_2_O/MeOH with 20 mM ammonium acetate as mobile phase at 0.6 mL/min and 25 °C. The ratio of the roflumilast content of the in vitro digested sample to the initial content was taken to represent its bioaccessibility. 

#### 2.2.5. Photostability Study

The spectra of roflumilast with or without CDs were obtained using a Jasco V-650 Spectrophotometer (Jasco, Spain) between 200 and 400 nm. Fluorescence spectra were obtained in a Shimazdu RF-6000 spectrofluorimeter (Shimadzu, Japan) equipped with thermostatically controlled cells. Excitation and emission bandwidths were both set at 2 nm. The excitation and emission wavelengths for roflumilast were 290 and 380 nm, respectively. The relative fluorescence intensity values were recorded at 25 °C. To avoid inner filter effects, 2 mm quartz cells were used. The concentration of roflumilast was fixed at 8 µM and the CD concentration was varied between 0 and 5 mM. All reagents were dissolved in 0.1 M pH 7 sodium-phosphate buffer 4% EtOH.

The HPLC-MS sample was prepared by exposing the sample at 290 nm for 30 min and analyzed using an Agilent 1200 series HPLC equipped with a TOF 6220 (acquisition range 100–1100) in positive mode. Six microliters of 8 µM irradiated roflumilast were injected using a 40/60 H2O/MeOH mixture with 20 mM ammonium acetate as mobile phase at 0.6 mL/min and 25 °C.

#### 2.2.6. Photodegradation Kinetic Study

The kinetic parameters of the reaction were obtained from consecutive reaction kinetics:

(6) $$A\;\overset{k1}{\to }\;I\;\overset{k2}{\to }P$$

where “A” was roflumilast, “I” the intermediate, “P” the product of the reaction and k_n_ the reaction rate constants. [I] and [P] can be expressed using the following equations:

(7) $$\left[I\right]=\left[A_{0}\right]\frac{k_{1}}{k_{2}-k_{1}}\left(e^{-k_{1}t}-e^{-k_{2}t}\right)$$

(8) $$\left[P\right]=\left[A_{0}\right]\left(1+\frac{1}{k_{2}-k_{1}}\right)\left(k_{2}e^{-k_{1}t}-k_{1}e^{-k_{2}t}\right)$$

The fluorescence signal is the product of each concentration and its fluorescence yield,

(9) F=Fi[I]+ Fp[P]

where Fi and Fp are the fluorescent intensities of each product. Adding Equations (7) and (8) in (9), a new equation can be obtained:

(10) $$F=Fi\left[A_{0}\right]\frac{k_{1}}{k_{2}-k_{1}}\left(e^{-k_{1}t}-e^{-k_{2}t}\right)\;+\;Fp\left[A_{0}\right]\left(1+\frac{1}{k_{2}-k_{1}}\right)\left(k_{2}e^{-k_{1}t}-k_{1}e^{-k_{2}t}\right)$$

#### 2.2.7. Data Analysis

The HPLC-MS experiments were carried out once, while the remaining experiments were carried out in triplicate. Graphical representations were made using SigmaPlot (Version 10.0, Systat, Germany) and GraphPad Prism (Version 5.03 GraphPad software, San Diego, CA, USA) was used for the kinetic fitting. A t-test was applied using Rstudio (version 0.99.878, Rstudio, Boston, MA, USA), fixing the significance level at P < 0.05. Other mathematical operations were carried out using wxMaxima software (version 12.04.0).

## 3. Results

### 3.1. Effect of CDs on the Solubility of Roflumilast

**Figure 2:**
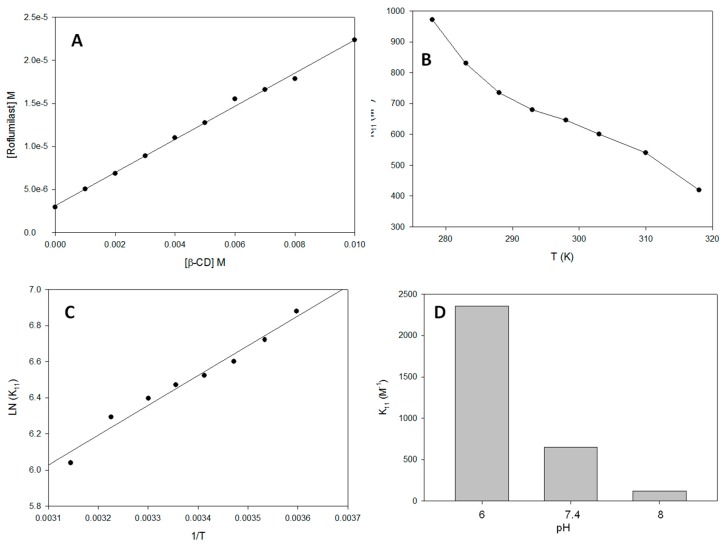
(**A**) Effect of β-CD (cyclodextrin) concentration on roflumilast solubility at pH 7.4 25 °C. (**B**) Effect of temperature on *K*_11_ values for roflumilast and β-CD complexes at pH 7.4. (**C**) Van’t Hoff plot. (**D**) Effect of pH on *K*_11_ values for roflumilast and β-CD complexes at 25 °C.

The first step was to study the effect of CDs on the solubility of roflumilast. The first candidate selected was β-CD because it has a similar inner cavity to HPβ-CD. Different tubes at a fixed roflumilast quantity (8 mg/mL) were prepared with increasing β-CD concentrations and mixed for 12 h. Figure 2A shows the variations in the apparent solubility of roflumilast in these conditions. A linear regression was carried out to obtain the slope for the *K*_11_ calculation, which gave a value of 646 ± 32 M^−1^. 

In order to increase the number of CDs evaluated with roflumilast, different natural (α- and γ-) and modified (HPβ-CD and Mβ-CD) CDs were used. The results (Table 1, Figure S1, Supplementary Materials) showing great variability. 

### 3.2. Effect of Temperature on Roflumilast and β-CD Complexation

One of the most important parameters that must be studied when using complexes as ingredients in the nutraceutical industry is the effect of temperature on the complexation mechanism, which must be tested at different temperatures. Figure 2B shows an inverse relationship between temperature and *K*_11_, while a direct relationship was obtained for S_int_ (the theoretical solubility obtained from the intercept regression) or S_0_. 

### 3.3. Thermodynamic Parameters for the Roflumilast- β-CD Complexes

The next step was to study the main thermodynamic parameters of the complexation process (*ΔH°*, *ΔS°* and *ΔG°* at 25 ± 0.2 °C) in order to study mechanistic aspects of the affinity of roflumilast for β-CD. For this, a van’t Hoff plot (Equation (4)) was used and the Ln *K*_11_ was plotted vs. 1/T. The data showed a lineal behavior, with a correlation coefficient higher than 0.96 (Figure 2C). Results showed the following values: ΔH° = –13.7 ± 0.9 KJ·mol^−1^, ΔS° = 7.4 ± 0.4 J mol^−1^ K^−1^ and ΔG° = −15.7 ± 0.8 KJ·mol^−1^.

### 3.4. Effect of pH on Complexation Constant of Roflumilast with β-CD 

Another important factor to bear in mind when a guest molecule/CD complex is used in the food or nutraceutical industry is the pH of the medium. Several authors have shown that the protonation state has a great influence on the encapsulation constants [11,14]. As shown in Figure 2D, *K*_11_ values are closely dependent on pH, passing from a value of 2356 ± 118 M^−1^ (when the medium pH is 6) to about 121 ± 6 M^−1^ (medium pH 8). 

### 3.5. Molecular Docking Simulations of Roflumilast/β-CD Complex 

**Figure 3:**
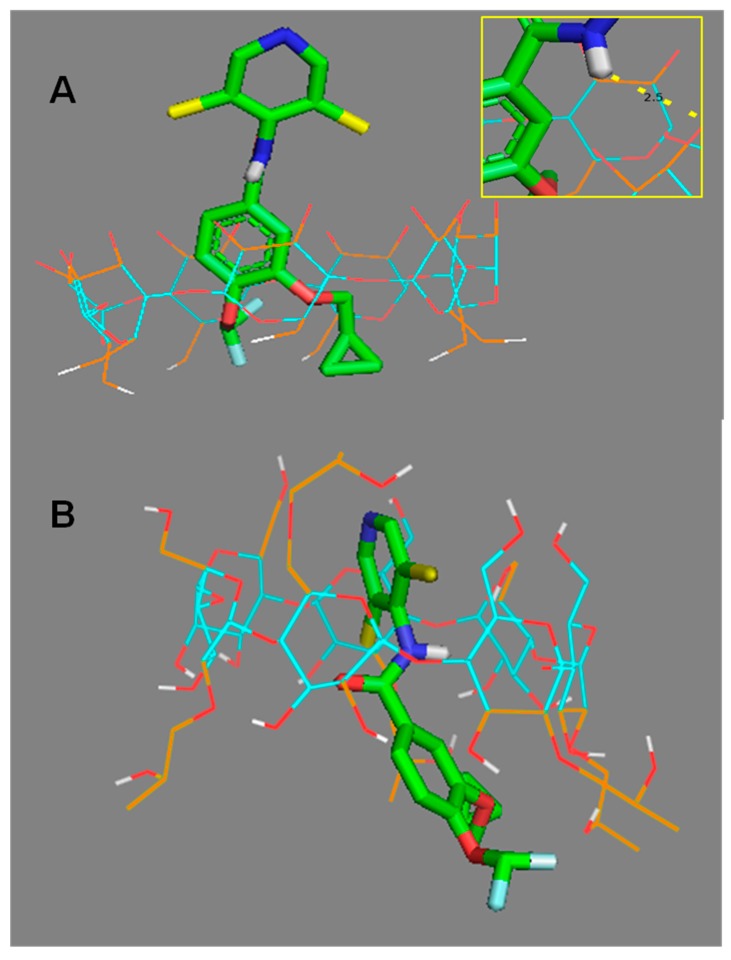
(**A**) Result for roflumilast/β-CD docking pose simulation, interactions are in yellow. Flexible atoms are coloured orange. *Insert*. Details of interaction. (**B**) Result for roflumilast/hydroxypropyl-β-CD (HPβ-CD) docking pose simulation, interactions are in yellow. Flexible atoms are coloured orange.

One of the most widely used techniques for predicting the host/guest interactions resulting from complexation with CDs is molecular docking [14,17]. After preparing the inputs, Vina software was used. The function score is a fast mathematical methods used to predict the strength of the non-covalent interaction (also referred to as binding affinity). The score for the roflumilast/β-CD complex was −8.9 and for HPβ-CD it was −6.9. Figure 3A shows the most probable pose of the Vina software. Furthermore, the amine atom was seen to be near (2.5 Ǻ) a hydroxyl part of CD. Figure 3B shows the vina pose for the roflumilast/HPβ-CD complex. The pose did not show hydrogen bonds.

### 3.6. Effect of CD Addition on the Roflumilast Digestion

**Figure 4:**
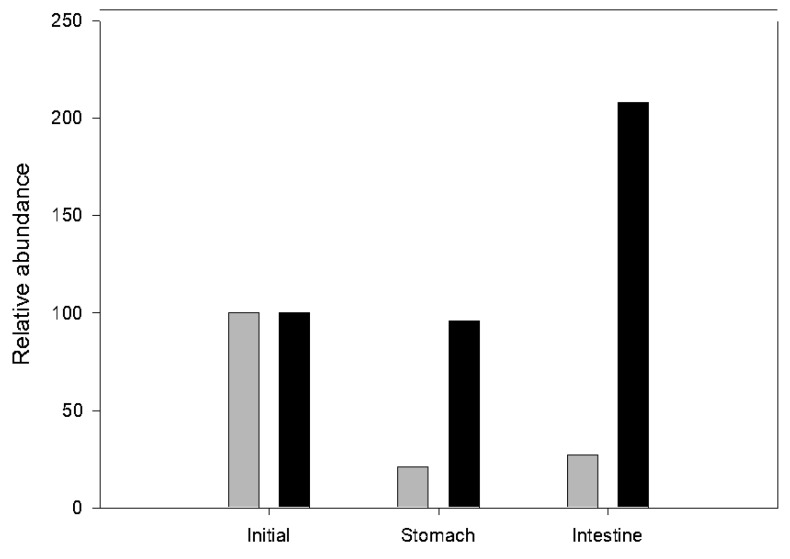
Relative abundance of roflumilast ions after stomach and intestine digestion without CD (gray) and with HPβ-CD (black). The data are normalized using initial roflumilast abundance.

Roflumilast is an orally administrated drug: for example DAXAS^®^ is administrated as a 500 mg tablet (1.24 µmols/day). Although the tablets are usually film coated, CDs could be considered as a carrier for oral administration (alone or in combination) of roflumilast. An experimental in vitro digestion was carried out for (i) roflumilast 0.2 mg/mL and (ii) roflumilast 0.2 mg/mL in the presence of 17.5 mg/mL of HPβ-CD in 0.24 L of final volume [24]. The CD concentration was 50% of the legally limited level [25]. In both cases roflumilast was supersaturated and only the soluble part was analyzed. The solution was filtered and diluted using phosphate-Na buffer pH 7.4 for analysis by LC-MS. Figure 4 shows the [Roflumilast-H]-abundance, and shows the abundance of the roflumilast ion and the adducts formed, normalized with their abundance at t_0_. The results showed that CDs not only protected roflumilast at pH 3, but also increased its solubility. Indeed, at pH 6.9, the solubility of roflumilast may increase explaining the increase in the roflumilast/CD sample and roflumilast alone. Roflumilast in acid (1 N HCl) conditions gives 3,5-dichloropyiridin-4-amine [26], and as HCl was used to adjust the pH in the in vitro stomach simulation maybe this products might appear.3,5-dichloropyiridin-4-amine sodium adduct was obtained with an abundance of 9320 without HPβ-CDs vs. 273 with HPβ-CD at the end of the digestion, a 34.13-fold increase. 

### 3.7. Effect of CD Addition on Roflumilast Photostability

**Figure 5:**
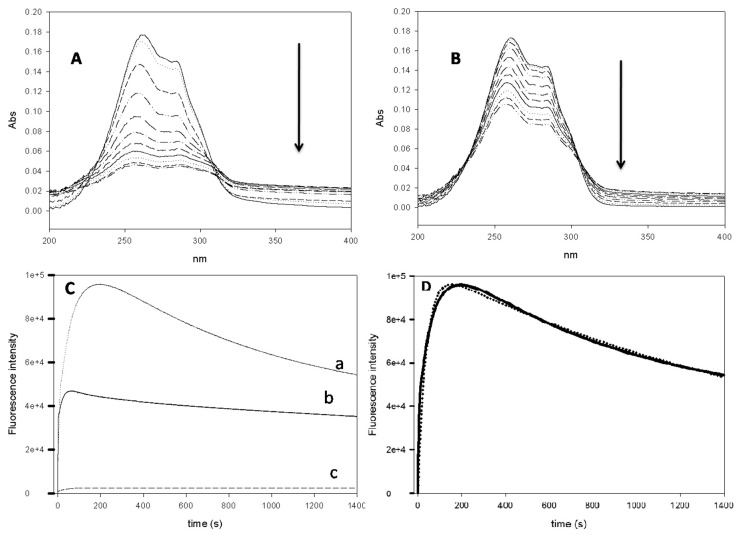
(**A**) Spectra of roflumilast 24 µM at 4% EtOH pH 7.4 and 25 °C every 2 minutes. (**B**) Spectra of roflumilast 24 µM at 4% EtOH with 5 mM β-CD at pH 7.4 and 25 °C every 2 minutes. (**C**) Fluorescence time-course for (a) roflumilast 8 µM at 4% EtOH pH 7.4 and 25 °C; (b) roflumilast 8 µM at 4% EtOH with 5 mM β-CD pH 7.4 and 25 °C and (c) roflumilast 8 µM at 4% EtOH pH 7.4 and 25 °C in N_2_ atmosphere. (**D**) Experimental fluorescence time course for roflumilast 8 µM at 4% EtOH pH 7.4 and 25 °C (represented by a line) and the fit (represented by dots).

The photostability of chlorine-containing drugs is well-documented [27]. For this reason, the photostability of complexed roflumilast was studied. Figure 5A,B shows the effect of the complexation on the consecutive absorbance signal. The decrease in absorbance was slower with HPβ-CD, reflecting the apparent greater protection of the drug. Moreover, at around 240 nm, an apparent isopectic point was observed, suggesting a destructive reaction is occurring. 

It is wellknown that the fluorine atom is fluorescent. So, the next step was to study the possible fluorescence of roflumilast. The results (Figure S2, Supplementary Materials) showed for the first time that roflumilast is fluorescent. The excitation and emission wavelengths for roflumilast were 290 nm and 380 nm, respectively. No phosphorescence was found. At these nm values, a time course analysis showed that the signal increased with time (25 min) (Figure 5C). The signal presented a maximum around 180 s of irradiation followed by a gradual decrease. Stopping the reaction before the maxima excitation did not return the drug to its original state. The reaction was slower in the presence of HPβ-CD, possibly due to the encapsulation and protection. Furthermore, an experiment without O_2_ (Inner N_2_ atmosphere) pointed to the absence of a fluorescence signal and no degradation of the drug. 

As the fluorescence signal did not return to basal state, an irreversible change must have occurred. An HPLC-MS analysis of the reaction showed an interesting peak at 367.0661 (Figure S3, Supplementary Materials), which presented exactly the mass as roflumilast without one chlorine atom. This product was suggested by Paul in (2015) as a fragment of their MS/MS studies [26]. 

### 3.8. Kinetics Analysis of the Reaction

In the previous section we explained the phenomena that occurred when roflumilast was irradiated. There seemed to be a consecutive reaction pathway where a substrate “A” reacts to give an unstable intermediate “I” and a stable “P” product. Our data suggest that the intermediate product must be more fluorescent than the final product; so, this signal could be used to obtain the kinetic parameters (k_1_ and k_2_) of the reaction by using Equation (10). Graphpad iterated the data and predicted the values of F_i_ = 1.28 · 10^10^ ± 6.4 10^8^ a.u., k_1_ = 2.86 · 10^−2^ ± 1.43 ± 10^−3^ s^−1^, k_2_ = 4.83 · 10^−4^± 2.42 ± 10^−5^ s^−1^ and F_p_ = 8.5 · 10^4^ ± 4 · 10^2^ a.u. with a R^2^ > 0.97 (Figure 5D). These results demonstrated that the conversion of roflumilast in the intermediate (A→ I) is the most important contribution to the fluorescence signal. Furthermore, k_1_ is 56 times higher than k_2_; So, the formation of the product would be the rate-determining step (RDS) of the reaction. With this in mind, a possible reaction scheme was formed (Figure S4, Supplementary Materials) where roflumilast, in the presence of oxygen and light, is irradiated giving an intermediate molecule, whose degradation releases one chlorine atom. 

## 4. Discussion

As can be seen in Figure 2A the solubility presented a behavior of a typical AL-type curve in the phase solubility diagram, with the exception of γ-CD that was AP-type curve (Figure S1, Supplementary Materials), perhaps this CD does not present an optimal diameter to solubilize roflumilast [28]. Moreover, the solubility with 8 mM was sixtimes higher than without β-CD A value that reflects the normal *K*_11_ values (between 50 and 2000 M^−1^ [29]) obtained with this technique. Of all the CDs tested, β-CD provided the highest values of *K*_11_ followed by Mβ-CD and HPβ-CD, the two modified CDs studied. It seems that the cavity of β-CD was optimal for encapsulating roflumilast; by contrast, the extra polarity of the hydroxypropyl substituent may prevent a better fit. The γ-CD presented a very close value to HPβ-CD (ANOVA, p ≈ 0.08), not statistically significant. The cavity of γ-CD may be sufficient for a good fit, although not optimal. Finally, α-CD presented the worst result. These observations demonstrated that the addition of CD to the solution can increase the apparent solubility of roflumilast, although, the complexation strength is not the same for all CDs. 

According to the temperature study, the encapsulation process is more efficient at low temperatures. The thermodynamic values obtained led to three main conclusions being drawn concerning the nature of the complexation of roflumilast by β-CD: (i) the process is *exothermic,* as deduced from the negative values obtained for enthalpy changes. This indicates the exothermic nature of the interaction processes of roflumilast with β-CD. This behavior is typical of hydrophobic interactions, van der Waals interactions and the displacement of water molecules from the cavity of β-CD or the formation of hydrogen bonds. (ii) The process presents a positive value for entropy changes possibly due to the water released from the β-CD cavity and/or the increase of hydrophobic interactions [30]; (iii) The process is spontaneous, as seen from the negative value obtained for the Gibbs free energy change (for the interactions that take place during the inclusion process at 25 ± 0.2 °C.

As regards pH, similar behavior was described by our group when the effect of pH on the *K*_11_ values of stilbenes-CD and fatty acid-CD complexes were studied [9,31]. The sharp decrease in the *K*_11_ value observed in Figure 2D coincides with the region where the roflumilast could be influenced by its pKa (8.74, pubchem). A possible cause for this pH-dependence of *K*_11_ would be the formation of a hydrogen bond between roflumilast and CD, since hydrogen bonding is one of the most important types of interaction in the stabilization of inclusion complexes [32,33]. The fact that the complexes between β-CD and roflumilast were more stable below 7.4 is of great interest for the industry, because lower CD concentrations are necessary for roflumilast to be administered.

The molecular docking score indicated that the encapsulation is spontaneous. The fact that the data correlated perfectly with the *K*_11_ value indicates that the predictions provided the essential interaction information between CD and roflumilast. The complete encapsulation of roflumilast by β-CD was observed, especially of the most hydrophobic part. The same profile was found for HPβ-CD. The chlorine atoms remained outside in the figure, perhaps because they are more hydrophilic than the other parts. Furthermore, the amine atom was seen to be near (2.5 Ǻ) a hydroxyl part of CD, where it probably contributed an important function to the stabilization of the complex HPβ-CD/roflumilastdid not show hydrogen bonds. Moreover, some interference with hydroxypropyl substituent (e.g., steric hindrance) could negatively affect the complexation.

After in vitrodigestion, our results indicate that the stomach part might be the most aggressive. For that reason, a film-coated tablet for the stomach part would be desirable if CDs are used. Even so, our results were quite good. The increase in roflumilast solubility demonstrated that CDs not only partially protect roflumilast during digestion, but increase its solubility and bioaccesibility, information that may be used to reformulate tablets because the same results can be obtained using less roflumilast.

The problem of photostability must be taken into account during the manufacture and administration of the drug. The use of CDs was seen to decrease the effect of light on roflumilast. The reaction was irreversible because no return to initial drug state was observed. These facts suggest that oxygen is crucial to the reaction mechanism, in contrasttochlorpromazine or hydrochloryhiazide [27]. The reaction released chlorine after light irradiation in the presence of oxygen, generating a fluorescent signal. This reaction can be prevented an inner atmosphere. CDs showed a protective effect on the reaction although the docking results suggested that the chlorine part remains outside the complex, perhaps because the protection of fluorine atoms prevents its irradiation.

## 5. Conclusions

The Higuchi-Connors method was used to obtain the values of *K*_11_ of natural and modified CDs with roflumilast; β-CD was the best CD tested with a value of 684.55 ± 34.23 M^−1^. The effect of temperature and pH on the encapsulation process was also evaluated. The *K*_11_ values increased at low temperatures. The thermodynamic parameters were also evaluated (ΔH° = −12.21 ± 0.6 KJ·mol^−1^, ΔS° = 12.93 ± 0.6 J·mol^−1^·K^−1^ and ΔG° _(25)_ = −15.73 ± 0.8 KJ·mol^−1^). The effect of pH was inversely proportional. These data agree with the increase in roflumilast solubility at higher temperature and pH values. Besides, molecular docking simulations were carried out to study their interactions. A high degree of correlation was observed between the computed score and experimental value. An in vitro digestion showed that CDs could protect this drug during digestion and improve its bioaccessibility. Finally, when the effect of CDs on the photostability of roflumilast was evaluated CDs were seen to reduce the photosensitivity of roflumilast. These changes originated a fluorescence signal, which is described for first time in this compound (λ excitation 290, λ emission 380). The fluorescence was seen to be dependent on O_2_ and to release one chlorine atom. Kinetic parameters calculated for a consecutive reaction showed that the formation of the intermediate is 53 times faster than the corresponding of the product formation and is responsible for the signal. This study not only completes the complexation study of roflumilast-CD, but also reveals the need to protect roflumilast from light and degradation.

## Figures and Tables

**Table 1:** Apparent *K*_11_ values and SD for Higuchi and Connors method (1:1 complex).

	B-CD	Mβ-CD	HPβ-CD	γ-CD	α-CD
K11 (M^−1^)	646	418	329	300	143
SD (±)	32	20	16	15	7

## Supplementary Materials

The following are available online at https://www.mdpi.com/2073-4360/11/5/801/s1, Figure S1. Effect of different CDs on Roflumilast solubility (25 °C pH 7). Figure S2. Emission spectrum of roflumilast 8 µM 4% EtOH at pH 7.4 (excitation 290). Figure S3. HPLC-MS peaks for Roflumilast (403.0428 ion, - - -) and Roflumilast without Cl (367.0661ion, __). Figure S4. Plausible simplified reaction mechanism.
