# Supplementary material for: A Way to Increase the Bioaccesibility and Photostability of Roflumilast, a COPD Treatment, by Cyclodextrin Monomers
Source: Polymers (Basel). 2019 May 4;11(5):801. doi: 10.3390/polym11050801 (PMC6571910; doi:10.3390/polym11050801)
Supplement: Supplementary file 1 [file polymers-11-00801-s001.pdf]

Supplementary Information

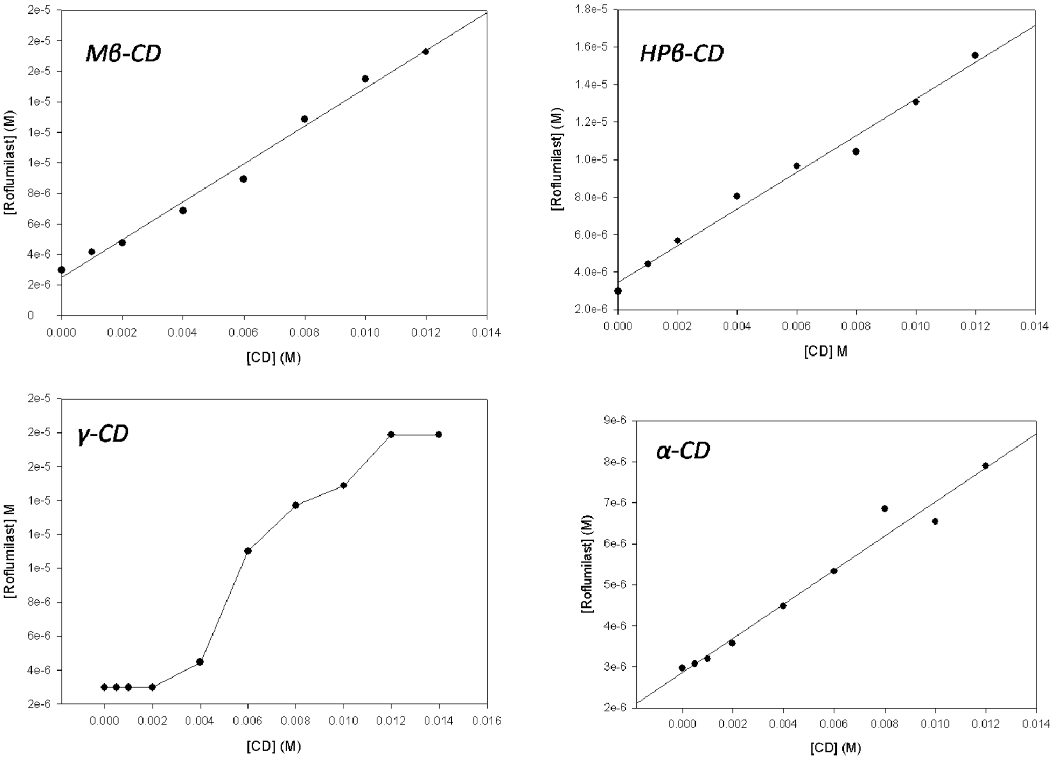

Figure S1. Effect of different CDs on Roflumilast solubility (25 °C pH 7).

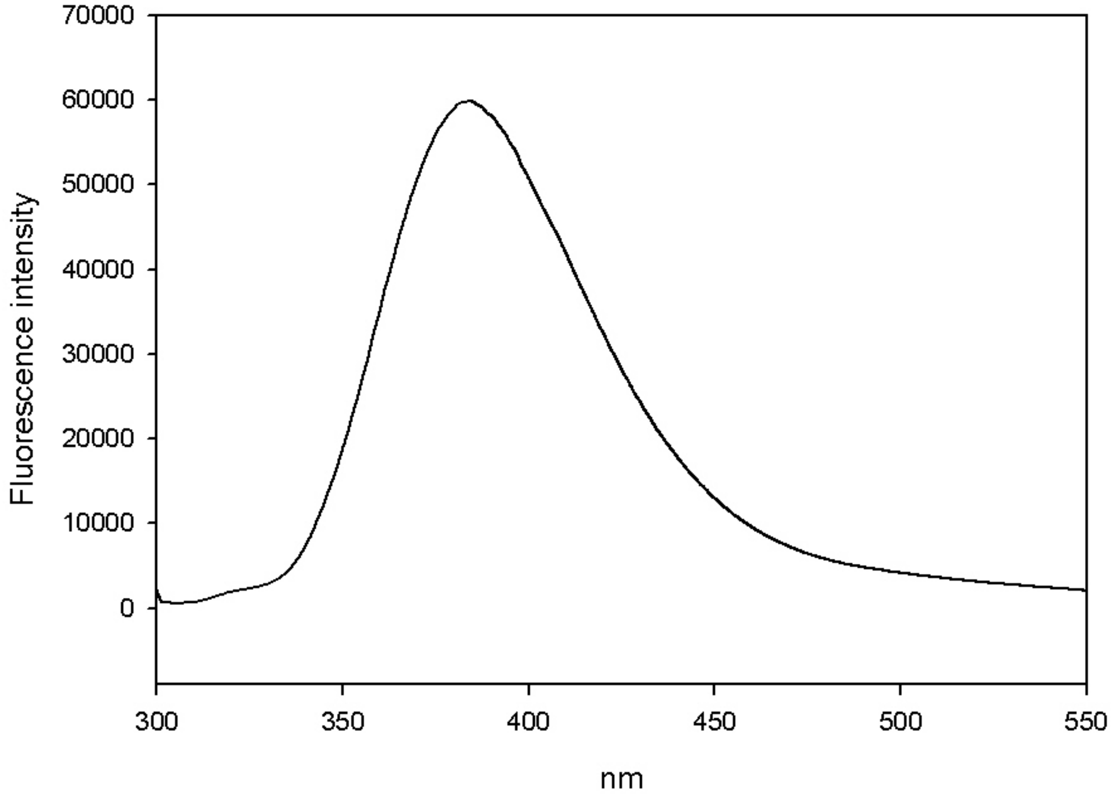

Figure S2. Emission spectrum of roflumilast 8 μM 4% EtOH at pH 7.4 (excitation 290).

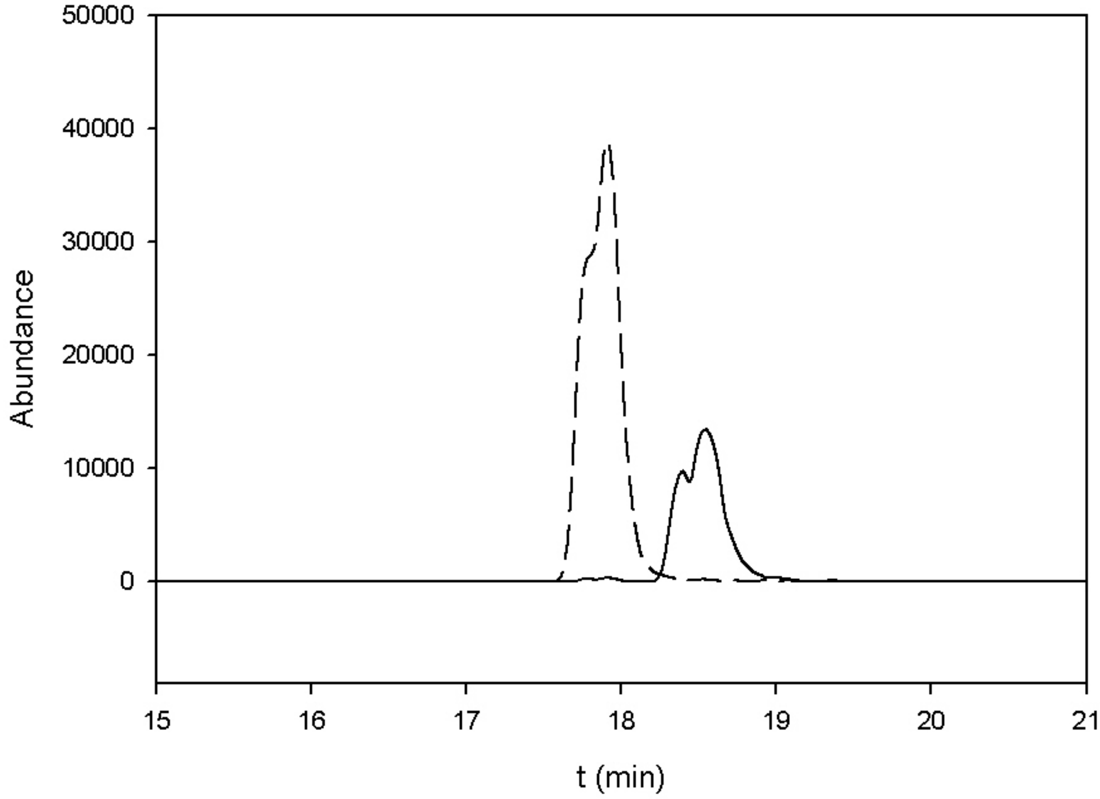

Figure **S3**. HPLC-MS peaks for Roflumilast (403.0428 ion, - - -) and Roflumilast without Cl (367.0661ion, __).

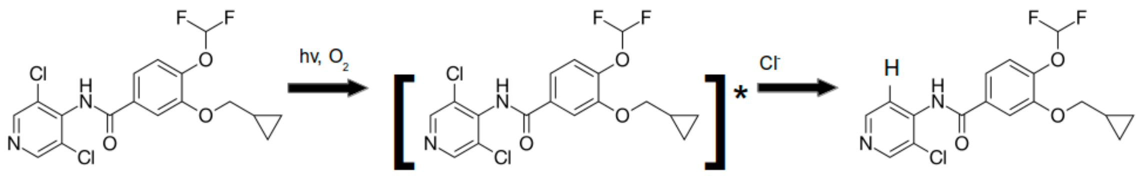

Figure **S4**. Plausible simplified reaction mechanism.
